# Hemorrhagic brain metastasis as the initial presentation of osteosarcoma: a rare case report

**DOI:** 10.1093/omcr/omaf138

**Published:** 2025-08-20

**Authors:** Salma El Aouadi, Rania Bouanane, Soukaina Bahha, Imane Iraqui, Asaad El Bakkari, F Z Laamrani, Youssef Omor, Rachida Latib, Sanae Amalik

**Affiliations:** Department of Radiology, National Institute of Oncology, Ibn Sina University Hospital Center, Avenue Allal El Fassi, Quartier des Orangers, Rabat 10000, Rabat-Salé-Kénitra Region, Morocco; Department of Radiology, National Institute of Oncology, Ibn Sina University Hospital Center, Avenue Allal El Fassi, Quartier des Orangers, Rabat 10000, Rabat-Salé-Kénitra Region, Morocco; Department of Radiology, National Institute of Oncology, Ibn Sina University Hospital Center, Avenue Allal El Fassi, Quartier des Orangers, Rabat 10000, Rabat-Salé-Kénitra Region, Morocco; Pathology Department, Ibn Sina University Hospital Center, Avenue Ibn Rochd, Agdal District, Rabat 10000, Rabat-Salé-Kénitra Region, Morocco; Department of Radiology, National Institute of Oncology, Ibn Sina University Hospital Center, Avenue Allal El Fassi, Quartier des Orangers, Rabat 10000, Rabat-Salé-Kénitra Region, Morocco; Department of Radiology, National Institute of Oncology, Ibn Sina University Hospital Center, Avenue Allal El Fassi, Quartier des Orangers, Rabat 10000, Rabat-Salé-Kénitra Region, Morocco; Department of Radiology, National Institute of Oncology, Ibn Sina University Hospital Center, Avenue Allal El Fassi, Quartier des Orangers, Rabat 10000, Rabat-Salé-Kénitra Region, Morocco; Department of Radiology, National Institute of Oncology, Ibn Sina University Hospital Center, Avenue Allal El Fassi, Quartier des Orangers, Rabat 10000, Rabat-Salé-Kénitra Region, Morocco; Department of Radiology, National Institute of Oncology, Ibn Sina University Hospital Center, Avenue Allal El Fassi, Quartier des Orangers, Rabat 10000, Rabat-Salé-Kénitra Region, Morocco

**Keywords:** osteosarcoma, hemorrhagic brain metastases, pulmonary metastases, pediatric oncology

## Abstract

Osteosarcoma is the most common malignant bone tumor in children and adolescents, with a predilection for long bones and frequent pulmonary metastases. Brain metastases are rare, occurring in 1.8% to 5.6% of cases, and hemorrhagic ones are exceptionally uncommon, with only a few cases documented in the literature. We report the case of a 16-year-old girl who presented with acute headache and seizures. Imaging revealed hemorrhagic brain and pulmonary metastases, and biopsy confirmed a primary femoral osteosarcoma. This case represents an exceptionally rare presentation of osteosarcoma and highlights its aggressive metastatic behavior.

## Introduction

Osteosarcomas are the most common primary malignant bone tumors in children and adolescents, typically arising in the metaphyses of long bones and spreading hematogenously to the lungs and other bones [1]. Brain metastases are rare and usually appear late in the disease course, particularly after relapse following treatment [1]. Their management requires a multidisciplinary approach, including corticosteroids to reduce peritumoral edema, surgical resection for accessible or symptomatic lesions, and radiation therapy, with systemic therapies tailored to the primary tumor [2]. Hemorrhagic brain metastasis as an initial presentation of osteosarcoma is exceptionally rare, posing significant diagnostic and therapeutic challenges. Increased awareness of such atypical presentations is essential, as early recognition can impact clinical management and prognosis.

**Figure 1:**
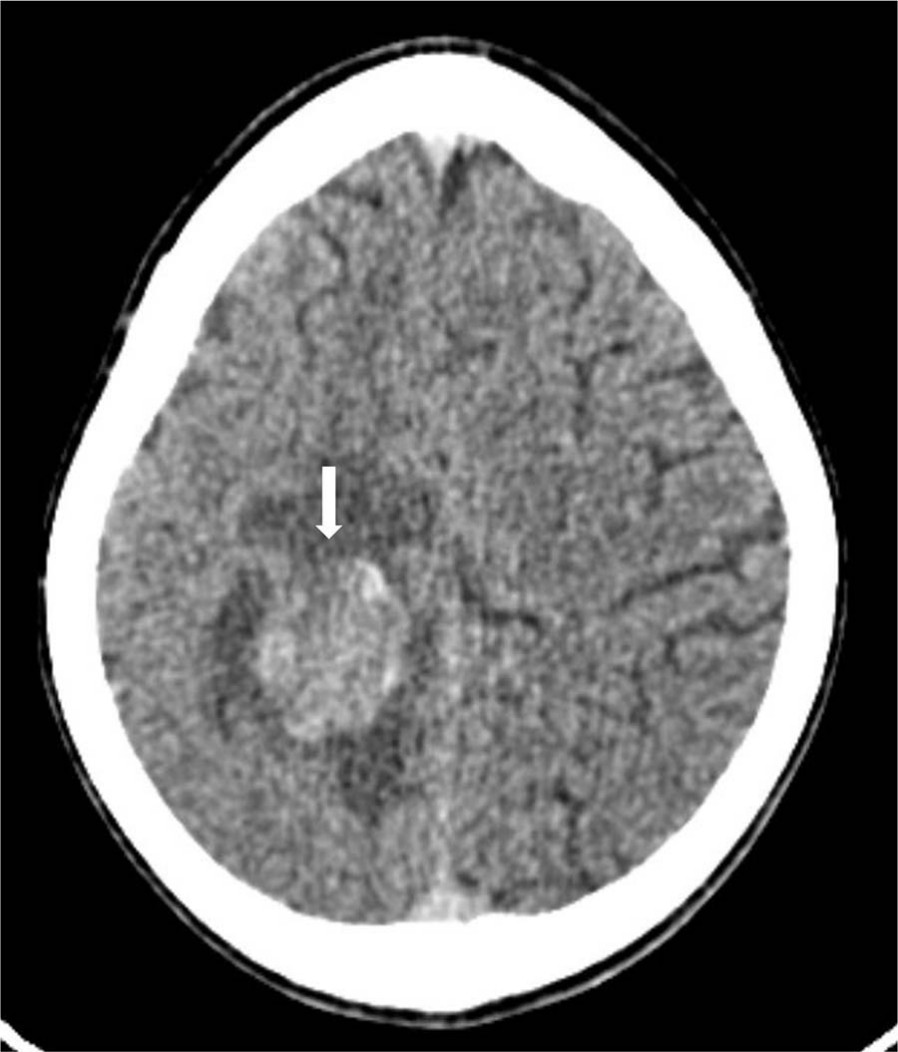
Axial brain CT scan showing an ovoid, well-defined right parietal lesion (arrow) with heterogeneous hyperdensity, surrounded by perilesional edema.

**Figure 2:**
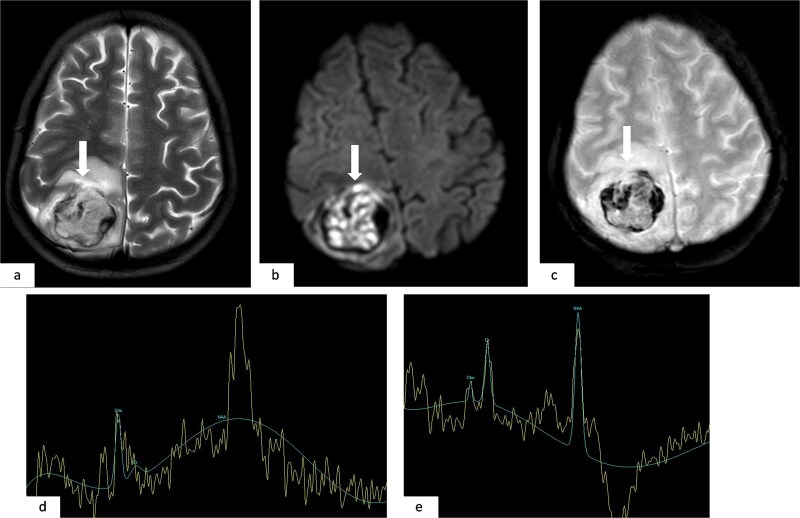
Axial T2-weighted MR image (a); axial diffusion-weighted MR image (b = 1000 s/mm^2^) (b); axial T2*-weighted gradient-echo MR image (c); multivoxel MR spectroscopy with long TE in the tumor (d) and in the peritumoral region (e). The right parietal lesion (arrows) shows intermediate signal intensity on T2-weighted imaging (a), high signal on diffusion-weighted imaging (b), and areas of low signal on T2*-weighted gradient-echo imaging (c), consistent with hemorrhagic foci. Multivoxel MR spectroscopy at long TE demonstrates an inverted choline/creatine ratio and decreased N-acetylaspartate (NAA) in the tumor region (d), with a normal metabolic profile in the peritumoral area (e).

## Case report

A 16-year-old Moroccan girl of Arab origin with a history of pain and swelling in the right lower limb presented to the emergency department with acute onset of headache, followed by continuous generalized tonic–clonic seizures lasting approximately 30 minutes without regaining consciousness between episodes. Urgent management with intravenous benzodiazepines was initiated.

Following stabilization, a contrast-enhanced brain computed tomography (CT) scan (Fig. 1) revealed a right parietal ovoid lesion with well-defined margins, heterogeneously hyperdense, and mild post-contrast enhancement. Brain magnetic resonance imaging (MRI) (Fig. 2) showed a well-circumscribed right parietal mass with intermediate signal on T2 and FLAIR-weighted images, diffusion restriction, and areas of intrinsic high signal on T1 and T2-weighted images, as well as low signal on T2*, consistent with hemorrhagic components. The lesion was surrounded by perilesional edema and demonstrated ring enhancement on post-contrast subtraction sequences. Multivoxel magnetic resonance spectroscopy (MRS) revealed a lactate and lipid peak on short time of echo (TE), decreased N-acetylaspartate (NAA), and an inverted choline/creatine ratio on long TE, with a normal spectrum in the peritumoral region, suggestive of a secondary brain lesion.

**Figure 3:**
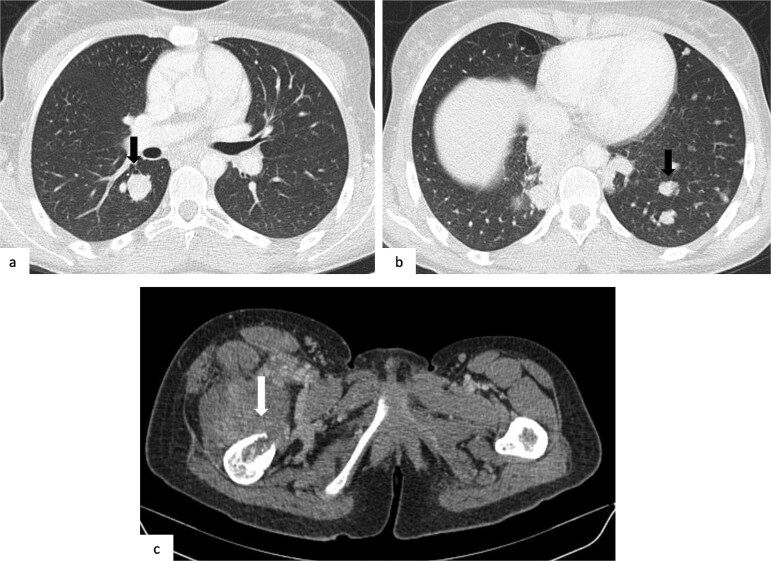
Axial contrast-enhanced CT images of the chest, abdomen, and pelvis: (a, b) thoracic CT scan in lung window showing multiple bilateral pulmonary masses and nodules, randomly distributed, dense with smooth contours (black arrows); (c) abdominopelvic CT scan revealing a lytic lesion centered on the right femoral neck, breaking through the cortex (white arrow).

**Figure 4:**
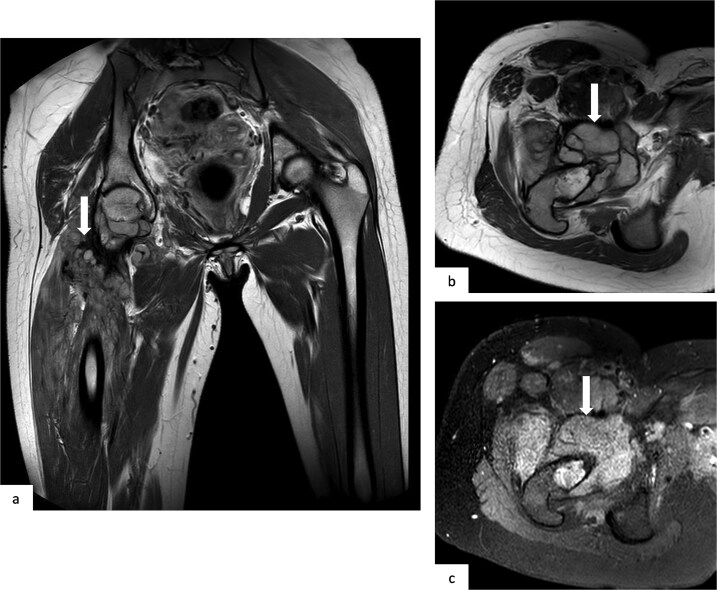
Coronal T2-weighted MR image (a), axial T2-weighted MR image (b), and axial T1-weighted fat-saturated MR image after gadolinium injection (c). MRI of the right thigh demonstrates a tumor centered on the femoral neck (arrows), showing high signal intensity on T2-weighted images (a,b) and heterogeneous enhancement after contrast administration (c). The lesion invades the coxofemoral joint and extends into the anterior muscle compartment.

A contrast-enhanced CT of the chest, abdomen, and pelvis (Fig. 3) revealed multiple bilateral pulmonary nodules with soft-tissue density and smooth margins, along with an aggressive lytic lesion centered on the right femoral neck breaking through the cortex and extending into the adjacent soft tissues. MRI of the thigh (Fig. 4) confirmed a large tumor centered on the right femoral neck, hyperintense on T2-weighted images, with diffusion restriction and heterogeneous enhancement.

**Figure 5:**
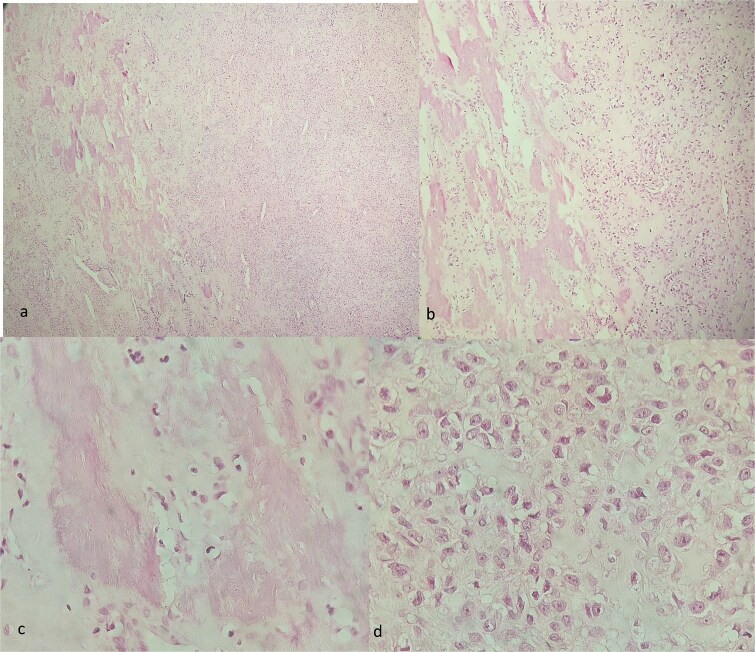
Histopathological images of conventional osteosarcoma at ×50 (a), x100 (b) and ×400 (c,d) magnification, showing pleomorphic tumor cells and the formation of immature osteoid.

A bone biopsy was performed, and histopathology confirmed a conventional osteosarcoma of the right femur (Fig. 5).

The patient was started on six cycles of IE protocol chemotherapy (Ifosfamide and Epirubicin), and whole-brain radiotherapy was administered with a total dose of 8 Gy. No surgical intervention was performed due to disseminated disease and the patient’s poor general condition. Despite treatment, follow-up imaging revealed progression, with enlargement of both pulmonary lesions and the primary femoral tumor. The patient’s condition worsened due to extensive metastases, resulting in respiratory failure from pulmonary nodules and neurological decline from brain involvement, which ultimately led to her death.

## Discussion

Osteosarcoma is the most prevalent malignant bone tumor in children and adolescents, characterized by an atypical osteoid matrix [3]. It typically affects the metaphysis of long bones but can also arise in the skull bones [3]. Brain metastases are rare in osteosarcoma, occurring in 1.8% to 5.6% of cases, often in conjunction with lung metastases [3]. Hemorrhagic brain metastases are even less common and may cause acute neurological symptoms such as headache, seizures, or focal deficits [4]. Diagnosis relies on CT and MRI to identify metastatic and hemorrhagic brain lesions. Treatment involves surgery, radiotherapy, and chemotherapy, but prognosis remains poor due to the tumor's aggressiveness and chemoresistance [2].

Although large clinical series report only a few cases of brain metastases in osteosarcoma [4, 5], our case is distinctive in that the hemorrhagic brain lesion was the initial presentation revealing the underlying primary tumor. As summarized in Table 1, only three similar cases have been described where hemorrhagic brain metastasis preceded diagnosis of osteosarcoma [6–8]. This highlights a reversed diagnostic sequence compared to the usual progression. Such atypical presentations emphasize the need for early brain imaging in young patients with unexplained intracerebral hemorrhage, to allow timely diagnosis and management [6, 7].

**Table 1:** Summary of clinical, radiological, and therapeutic characteristics of reported cases of hemorrhagic brain metastasis revealing osteosarcoma.

Case	Age/Sex	Primary tumor site	Initial presentation	Brain metastasis as initial finding	Pulmonary metastases	Imaging findings	Treatment	Outcome
Our Case	16/Female	Right femur	Seizures	Yes	Yes	Hemorrhagic lesion in right parietal lobe with surrounding edema	Radiotherapy, chemotherapy	Deceased
**Dwivedi et al., 2011** [6]	22/Female	Right iliac bone	Sudden right-sided weakness, transient loss of consciousness	Yes	Yes	Multiple hematomas in frontal lobe with significant edema; CT revealed lytic lesion in right iliac bone	Supportive care	Not specified
**Niazi et al., 2009** [7]	16/Male	Right femur metaphysis	Acute neurological deterioration due to posterior fossa hemorrhage	Yes	Yes	Hemorrhagic cerebellar lesion; MRI showed mass with surrounding edema	Surgical resection	Deceased 6 days post-surgery
**Menassa et al., 1997** [8]	Not specified	Left fibula	Symptoms of intracranial hypertension	Yes	No	Multiple intracranial hemorrhagic lesions; MRI suggested brain metastases	Not specified	Not specified

The primary dissemination pathway is hematogenous, as bones lack lymphatic vessels. Lung metastases often serve as a source of tumor emboli that reach the brain [3, 7]. The fragile and abnormal vascularization of these metastases predisposes them to hemorrhage [3, 7]. They typically localize at the gray-white matter junction within the anterior circulation, mostly in the cerebral cortex, although cerebellar involvement has also been reported [7].

The diagnosis of brain metastases is primarily established through neuroimaging modalities such as CT and MRI [9]. These metastases often appear as mass lesions with surrounding vasogenic edema, showing ring, nodular, or solid enhancement with contrast [9]. However, they can mimic other hypervascular lesions, including primary brain tumors like glioblastoma multiforme and meningiomas, complicating the differential diagnosis [7]. Glioblastomas typically exhibit infiltrative growth and distinctive metabolic patterns on MR spectroscopy, while meningiomas are usually extra-axial with dural attachment and characteristic imaging features [7, 9]. Other hemorrhagic brain metastases, such as those from melanoma or renal cell carcinoma, share a risk of bleeding but differ in clinical context and primary origin [6].

A multimodal approach is frequently employed in managing brain metastases in osteosarcoma patients [2]. Treatment strategies may include surgical resection in cases of solitary lesions or those associated with life-threatening intracranial hemorrhage [2]. Postoperative radiotherapy, either as stereotactic radiosurgery or whole-brain radiotherapy, is often employed to improve local control and reduce the risk of recurrence [2]. Systemic chemotherapy may be considered for addressing residual metastatic disease; however, its efficacy in treating brain metastases is limited due to the blood–brain barrier and the chemoresistant nature of osteosarcoma [2]. Treatment decisions should consider the patient’s condition, systemic disease extent, and risk–benefit balance.

The prognosis for patients with intracranial metastases is generally poor, with brain metastases occurring approximately 20 months after the initial diagnosis in soft tissue sarcomas and overall survival lasting only a few months [10]. These metastases often represent the final stage of disease progression [10].

This case illustrates an unusual initial presentation of pediatric osteosarcoma, with hemorrhagic brain metastasis rarely described in the literature. It emphasizes the need for early brain imaging in patients presenting with neurological symptoms and expands current knowledge of the diagnostic and therapeutic challenges posed by such uncommon metastases.

## Conclusion

Brain metastases, particularly hemorrhagic ones, are an exceptionally rare complication of osteosarcoma, often reflecting advanced disease and poor prognosis. This case highlights the importance of recognizing this rare presentation to guide timely diagnosis and management. Moreover, it underscores the need for early brain imaging in pediatric osteosarcoma patients presenting with neurological symptoms, even at the time of initial diagnosis, to facilitate prompt intervention and potentially improve outcomes.

## Guarantor

Dr El Aouadi Salma.
